# Activity Responses of *Rana forreri* and *Rhinella horribilis* Tadpoles to Predation Cues

**DOI:** 10.1093/icb/icag035

**Published:** 2026-05-08

**Authors:** Dariana González-Aguilar, Juan G Abarca, Yash Sondhi, Pablo Allen, José Miguel Chaves-Fallas

**Affiliations:** Department of Integrative Biology, Harvard University, Cambridge, Massachusetts 02138, USA; Unidad de Microbiología Médico Veterinaria, Servicio Nacional de Salud Animal (SENASA), Heredia 40104, Costa Rica; Alianza Nacional de Conservación de Anfibios y Reptiles (ANCAR), Alajuela 20106, Costa Rica; Department of Biology, Case Western Reserve University, Cleveland, Ohio 44106, USA; Council on International Educational Exchange (CIEE)-Monteverde, Puntarenas 61201, Costa Rica; Council on International Educational Exchange (CIEE)-Monteverde, Puntarenas 61201, Costa Rica; Escuela de Ciencias Biológicas, Universidad Latina de Costa Rica, San José 11501, Costa Rica

## Abstract

Tadpoles must rapidly detect and respond to predation cues to survive the most vulnerable stage of anuran development. Although previous studies have examined the effects of visual or chemical cues on tadpole behavior, none have compared the effects of multiple predator-associated sensory stimuli across species. Here, we examined the responses of two neotropical tadpole species, the Forrer’s leopard frog *Rana forreri* (non-toxic), and the cane toad *Rhinella horribilis* (toxic), to visual, chemical, and auditory predation cues. Tadpoles were exposed to simulated bird shadows (visual cue), belostomatid-conditioned water (chemical cue), and calls of the Green Kingfisher predator, *Chloroceryle americana* (auditory cue). We recorded tadpole activity for 4 min prior to, 1 min during, and 4 min following treatment exposure using portable locomotion activity monitors (pLAMs). *Rana forreri* exhibited increased activity in response to auditory cues, whereas chemical and visual cues did not produce significant changes in activity levels relative to the control. Conversely, *R. horribilis* showed no significant differences in activity level across treatments. These findings suggest that antipredation responses vary in their sensitivity to sensory modality and may be shaped by species-specific defensive strategies. In particular, chemical defense may reduce reliance on immediate behavioral responses, while non-toxic species may exhibit stronger responsiveness to specific sensory cues. Together, our results highlight the selective prioritization of sensory information in shaping antipredation behavior in larval anurans.

## Abstract (es)

Los renacuajos deben detectar y responder rápidamente a las amenazas de depredación para sobrevivir a la etapa más vulnerable del desarrollo de los anuros. Aunque estudios previos han examinado los efectos de señales visuales o químicas en el comportamiento de los renacuajos, ninguno ha comparado los efectos de múltiples estímulos sensoriales asociados a depredadores entre especies. En este estudio, evaluamos las respuestas de dos especies neotropicales de renacuajos, la rana leopardo de Forrer, *Rana forreri* (no tóxica), y el sapo de caña, *Rhinella horribilis* (tóxico), ante señales de depredación visuales, químicas y auditivas. Los renacuajos fueron expuestos a sombras simuladas de aves (señal visual), agua acondicionada por belostomátidos (señal química) y vocalizaciones del martín pescador verde, *Chloroceryle americana* (señal auditiva). Registramos la actividad de los renacuajos durante cuatro minutos previos, un minuto durante, y cuatro minutos posteriores a la exposición al tratamiento utilizando monitores portátiles de actividad locomotora (pLAMs).* Rana forreri* mostró un aumento en la actividad en respuesta a las señales auditivas, mientras que las señales químicas y visuales no produjeron cambios significativos en los niveles de actividad en comparación con el control. En contraste, *R. horribilis* no mostró diferencias significativas en los niveles de actividad entre tratamientos. Estos resultados sugieren que las respuestas antipredatorias varían en su sensibilidad según la modalidad sensorial y pueden estar determinadas por estrategias defensivas específicas de cada especie. En particular, la defensa química puede reducir la dependencia de respuestas conductuales inmediatas, mientras que las especies no tóxicas pueden mostrar una mayor sensibilidad a señales sensoriales específicas. En conjunto, nuestros hallazgos destacan la priorización selectiva de la información sensorial en la configuración del comportamiento antipredatorio en anuros en etapa larvaria.

## Introduction

Prey organisms face the fundamental challenge of detecting predation threats in complex, information-rich environments. For aquatic prey in particular, multimodal sensory integration, or how organisms prioritize and interpret different forms of sensory information, plays a central role in shaping survival and structuring predator-prey dynamics (Lima and Dill 1990; Ferrari et al. 2010). Understanding the sensory mechanisms underlying threat detection and escape behavior therefore provides critical insight into how animals make decisions under risk and how these decisions scale up to influence ecological interactions.

The tadpole stage of anuran development represents an ideal system for investigating multimodal sensory integration under predation risk. During this stage, tadpoles experience heightened susceptibility to predation and must rapidly detect and respond to predator cues in their environment to survive one of the most vulnerable periods of their life cycle (Laurila and Aho 1997). Many species learn to recognize said threats through exposure to sensory signals, such as moving shadows above water, predator vocalizations, and dissolved chemicals in water, that are associated with potential predators (Schmidt and Amézquita 2001). However, the relative prioritization of these different sensory modalities remains poorly understood, particularly across taxa that differ in predation pressure and defensive strategies.

In tadpoles, responses to predation cues typically manifest as behavioral adjustments in activity level or spatial avoidance of perceived threats (Schmidt and Amézquita 2001; Eterovick et al. 2020). Antipredation responses can include both quantitative and qualitative changes in behavior, such as reduced movement, refuge-seeking, spatial avoidance, or rapid high-speed swimming responses (Jungblut et al. 2025). Among these, reduced activity is the most widely documented and commonly used proxy for antipredation behavior, as decreased movement can reduce detection by visually or mechanosensory-oriented predators (Feminella and Hawkins 1994; Martel and Dill 1995; Schmidt and Amézquita 2001; Sansom et al. 2009).

These behavioral changes arise through the integration and interpretation of sensory stimuli. Visual sensitivity, although limited during early developmental stages, increases as tadpoles mature, allowing them to discriminate changes in light intensity and detect approaching predators, which can in turn trigger shifts in activity (McClure et al. 2009; Ding et al. 2014; Butler et al. 2024; Flores et al. 2024). Similarly, exposure to predator-derived kairomones, or chemical cues, often results in reduced movement or refuge-seeking behaviors in larval anurans (Feminella and Hawkins 1994; Flores et al. 2024). Auditory cues, including anthropogenic sounds, can also alter tadpole behavior, although their role in predator recognition remains comparatively understudied (Castaneda et al. 2020).

Despite substantial research on visual and chemical cue detection (Feminella and Hawkins 1994; Ding et al. 2014; Szabo et al. 2021; Flores et al. 2024), major gaps remain in our understanding of how tadpoles integrate multiple sensory modalities when assessing predation risk. To our knowledge, no study has experimentally compared tadpole responses to visual, chemical, and auditory predator-associated cues within a single framework. In addition, the extent to which these defensive behaviors vary between toxic and non-toxic tadpoles remains unclear. Because chemical defense is associated with reduced predation pressure in anurans (Jiang et al. 2023), toxic species may rely less on immediate escape behaviors than non-toxic species. This expectation is supported by comparative studies in other taxa, including aposematic butterflies, in which chemically defended species exhibit reduced activity levels and weaker or delayed escape responses compared to their non-toxic counterparts (Ruxton et al. 2018).

Here, we investigate how tadpoles of the non-toxic Forrer’s leopard frog (*Rana forreri*, Ranidae) and toxic cane toad (*Rhinella horribilis*, Bufonidae) change their activity levels in response to visual, chemical, and auditory predation cues. Specifically, we aim to evaluate whether different sensory cues vary in their potential to induce antipredation behavior in tadpoles and whether toxicity influences the magnitude of these responses. Because tadpoles inhabit dynamic aquatic environments with diverse predator assemblages, understanding how they prioritize and integrate multiple types of sensory information provides critical insight into the processes structuring predator-prey interactions during early life stages.

## Materials and methods

### Study species

**Fig. 1:**
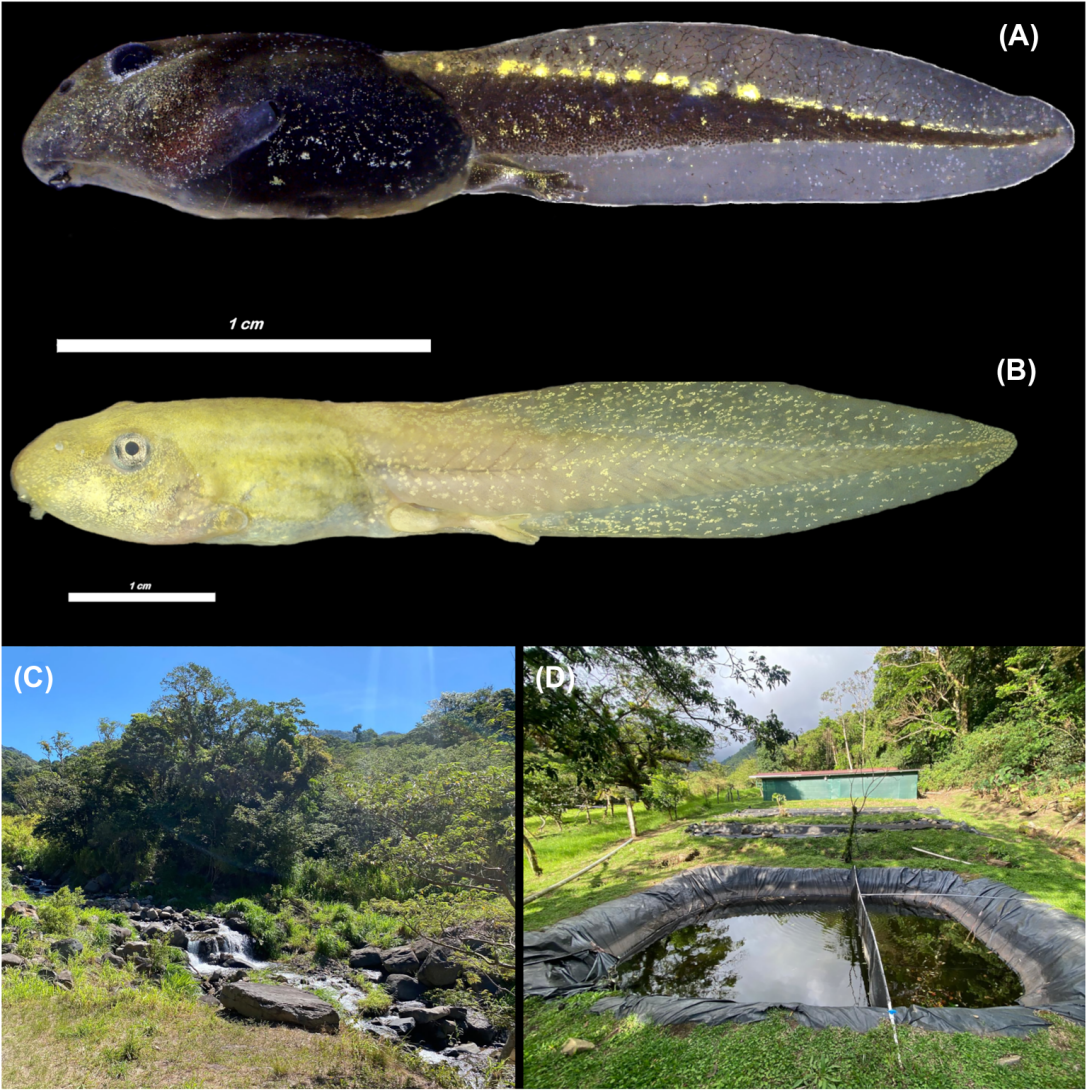
(A) Tadpoles of *Rhinella horribilis*, and (B) *Rana forreri*, and their respective (C) San Luis River and (D) CIEE-Monteverde pond habitats, in Monteverde, Costa Rica. Tadpole photos taken by Juan G. Abarca and habitat photos taken by Dariana González-Aguilar.

The cane toad, *Rhinella horribilis* (Bufonidae), ranges from the southern United States to Peru (Frost et al. 2006). In Costa Rica, it is abundant near human settlements, occurring from sea level to approximately 1600 m.a.s.l. (Leenders 2016). Larvae of *R. horribilis* inhabit slow-moving aquatic habitats and possess bufotoxins that remain active throughout all life stages (Hayes et al. 2009) (Fig. 1A).

The Forrer’s leopard frog, *Rana forreri* (Ranidae), ranges from southwestern Mexico to southwestern Costa Rica. This non-toxic frog species occurs in low-to-mid elevation regions of the Pacific side of Costa Rica, where it typically inhabits grassy edges of swamps, ponds, and streams (Leenders 2016; Leenders 2023; Chambers et al. 2025) (Fig. 1B).

Predators of tadpoles include herons and kingfishers, medium-sized piscivorous birds that forage along riparian corridors (Betts and Betts 1977; Castanho 1994; Čech and Čech 2015; Terres 1968), as well as belostomatid hemipterans (Belostomatidae), which capture aquatic prey using specialized raptorial forelegs (Schmidt and Amézquita 2001). Belostomatids are largely unaffected by cane toad bufotoxins (Cabrera-Guzmán et al. 2012), and avian predators generally exhibit a higher tolerance to these toxins than many vertebrate predators (Beckmann and Shine 2009).

### Tadpole collection

Fieldwork was conducted in Los Altos de San Luis, Monteverde, Puntarenas, Costa Rica (Lat 10°16′22.44″N, long 84°49′27.12″W). Data were collected during the dry season, between April 15 and 29, 2024. We collected a total of 120 tadpoles (60 per species) in sets of 20 individuals. Three sets of *R. forreri* tadpoles were collected from discrete pools along the San Luis River (Fig. 1C), and three sets of *R. horribilis* tadpoles were collected from an artificial pond on the CIEE-Monteverde campus (Fig. 1D); alternative ponds supporting *R. horribilis* were not identified during preliminary surveys in the study region. *Rana forreri* tadpoles were sampled from multiple pools to reduce the likelihood of collecting closely related individuals and to reduce the potential confounding effects of genetic relatedness on behavior. Individuals were collected using an aquarium net and transferred to 1L jars containing water from the same collection site. Four adult belostomatids were also collected from the San Luis River for use in producing chemical cues.

### Experimental set-up

All tadpoles were examined under a dissecting scope after collection to confirm species identity. To minimize variability in sensory capacity, each batch consisted exclusively of individuals at Gosner stages 30–34, corresponding to mid-larval development on the generalized staging system described by Gosner (1960).

From each batch of 20 tadpoles, four groups of five individuals were randomly assigned to separate plastic containers (20 × 14 cm). Containers were filled with 800 mL of site water to a depth of 3 cm and contained no leaf litter or substrate. This density of tadpoles (~6.25 individuals/L) is consistent with established protocols for anuran behavioral experiments and falls within the range reported for natural populations, particularly in temporary or spatially constrained habitats (Alford et al. 1995; Browne et al. 2003; Melvin and Houlahan 2014). Tadpoles were acclimated for 12 h prior to experimentation under controlled laboratory conditions (20–22°C and a natural 12:12 h light cycle, with natural daylight supplemented by LED full-spectrum lighting). Each container received one pinch of commercial fish food per day and was gently aerated using aquarium air pumps. These conditions were maintained throughout the experimentation period.

Belostomatids were held in 150 mL of water without food for 24 h to allow their kairomones, or predator-derived chemical cues, to accumulate (Mirza et al. 2006; Szabo et al. 2021). The resulting predator-conditioned water served as the chemical cue in experimental trials.

### Effect of predation cues on tadpole activity levels

Tadpole activity was quantified using portable locomotion activity monitors (pLAMs), which detect movement by measuring pixel differences between successive video frames, as described by Sondhi et al. (2022). The devices logged a “movement event” (ME) when a shift in an organism’s position exceeded a threshold of 200 pixels. Because MEs are based on positional changes between frames, they reflect the frequency of detectable movements rather than their magnitude (e.g., distance or speed). As such, MEs provide a proxy for overall locomotor activity, a widely used indicator of antipredation behavior in tadpoles, as both reductions (freezing) and increases (escape responses) in movement are commonly interpreted as responses to predation risk (Schmidt and Amézquita 2001; McClure et al. 2009; Ferrari et al. 2012; Jungblut et al. 2025). This approach prioritizes quantifying immediate changes in activity rather than detailed movement types or spatial patterns, which were beyond the scope of this study. Furthermore, because pLAMs quantify activity at the container level, all measurements represent aggregate locomotor activity of five tadpoles per container. This group-level metric is standard in pLAM-based studies and is well-suited for assessing population-level behavioral responses (Sondhi et al. 2022).

Four pLAM units were mounted on vertical stands positioned 15 cm above their respective tadpole containers. Devices were programmed to record tadpole activity for 4 min before, 1 min during, and 4 min after stimulus introduction. This observation period was selected to capture both baseline activity and the immediate and short-term behavioral responses to predation cues while minimizing potential habituation. Similar pre- and post-stimulus durations have been used in previous studies of tadpole antipredation behavior (Ferrari et al. 2012; Gazzola et al. 2024; Jungblut et al. 2025), which demonstrate that behavioral responses such as freezing and escape movements occur rapidly and are typically expressed within minutes of cue detection. Experiments were conducted after the acclimation period using the following treatments:

*Visual stimulus:* a 20 × 10 cm cardboard silhouette of a bird in flight was passed horizontally over the experimental container for 1 min. To standardize shadow intensity and contrast, the silhouette was maintained 15 cm above the container across all trials by aligning a ruler parallel to each pLAM unit and guiding the silhouette along this reference plane. Thirty passes were conducted per trial at a constant speed. Trials were performed at peak daylight hours to maximize shadow visibility (McClure et al. 2009).

*Chemical stimulus*: Thirty drops (~1.5 mL) of belostomatid-conditioned water were added to the center of the container over 1 min (one drop every two seconds). Previous studies have demonstrated that the addition of small volumes (~0.002 mL of chemical cue per mL of tadpole water) of predator-conditioned water is sufficient to elicit significant behavioral responses in tadpoles (Polo-Cavia et al. 2010).

*Auditory stimulus*: a recording of a Green Kingfisher (*Chloroceryle americana*, Alcedinidae) call from Puntarenas, Costa Rica (Merlin Sound ID database; Cornell Lab of Ornithology 2024) was played for 1 min from a standard iPhone 11 speaker positioned 5 cm from the container. Playback sound ranged from 33.8 to 71.8 dBA.

*Control*: control groups were recorded under identical conditions without stimulus introduction. This consistent timing ensured that any observed differences in activity between treatments could be attributed to cue-specific responses.

Each treatment was replicated three times per species, with one container per replicate (*n* = 3 containers per treatment per species). Across four treatments, this resulted in 12 containers per species and 24 total experimental units. No tadpole was used in more than one trial, ensuring independence of experimental results and reducing the potential for habituation effects. Statistical analyses were conducted using containers as the unit of replication, reflecting the group-level nature of the activity data.

### Statistical analysis

Activity data were analyzed separately by species and by temporal period, defined as pre-stimulus (4 min prior to cue exposure) and post-stimulus activity (1 min during plus 4 min after exposure). Combining the during- and post-stimulation periods into a single post-stimulus measure allowed us to capture both immediate and sustained antipredation responses, which together provide a more ecologically realistic representation of antipredation behavior. In natural conditions, predation risk often extends beyond the moment of initial detection, and maintaining altered activity after a perceived threat may be critical for survival. Consistent with this, previous studies have shown that tadpole physiological and behavioral responses to predator cues occur rapidly and persist for several minutes following cue exposure (Ferrari et al. 2012; Bennet et al. 2016; Jungblut et al. 2025).

For temporal period comparisons, individuals from all three predation cue treatments were pooled within each species to test for overall stimulus-induced changes in activity. Pre-stimulus activity levels of *R. horribilis* and *R. forreri* were compared using an independent samples *t*-test, and within-species differences between pre-stimulus and post-stimulus MEs were evaluated with paired samples *t*-tests, as measurements were taken from the same tadpoles. For each test, we report the *t-*value, degrees of freedom, and *P*-value in parentheses and the mean ($\bar{x}$) and standard deviation (*SD*) for the groups being compared.

To evaluate the effect of predation cue treatments, analyses were conducted separately by species to account for inherent differences in baseline activity and ecological traits. Given the relatively small sample size (*n* = 3 containers per treatment per species), this stepwise approach avoided overparameterization while providing a clear framework for testing species-specific responses to predation cues. We used parametric or non-parametric Analysis of Variance (ANOVA) tests depending on whether model assumptions were met. Assumptions of normality and equal variances were assessed using Shapiro-Wilk and Levene’s tests, respectively. *Rana forreri* activity data violated normality assumptions in the control (*P =* 0.004) and auditory (*P =* 0.026) groups but met homoscedasticity assumptions (*P =* 0.472); therefore, activity levels across treatments were compared using a Kruskal-Wallis test followed by Dunn’s post hoc tests with multiple-comparison corrections. Activity data for *R. horribilis* met assumptions of normality (*P >* 0.05 for all groups) and homoscedasticity (*P =* 0.219) and were thus analyzed using a one-way ANOVA. We report the corresponding *H-, F-*, and *P-*values of these tests. All statistical analyses were conducted in R version 4.2.2 (R Core Team 2024).

## Results

### Pre- versus post-stimulation activity levels

**Fig. 2:**
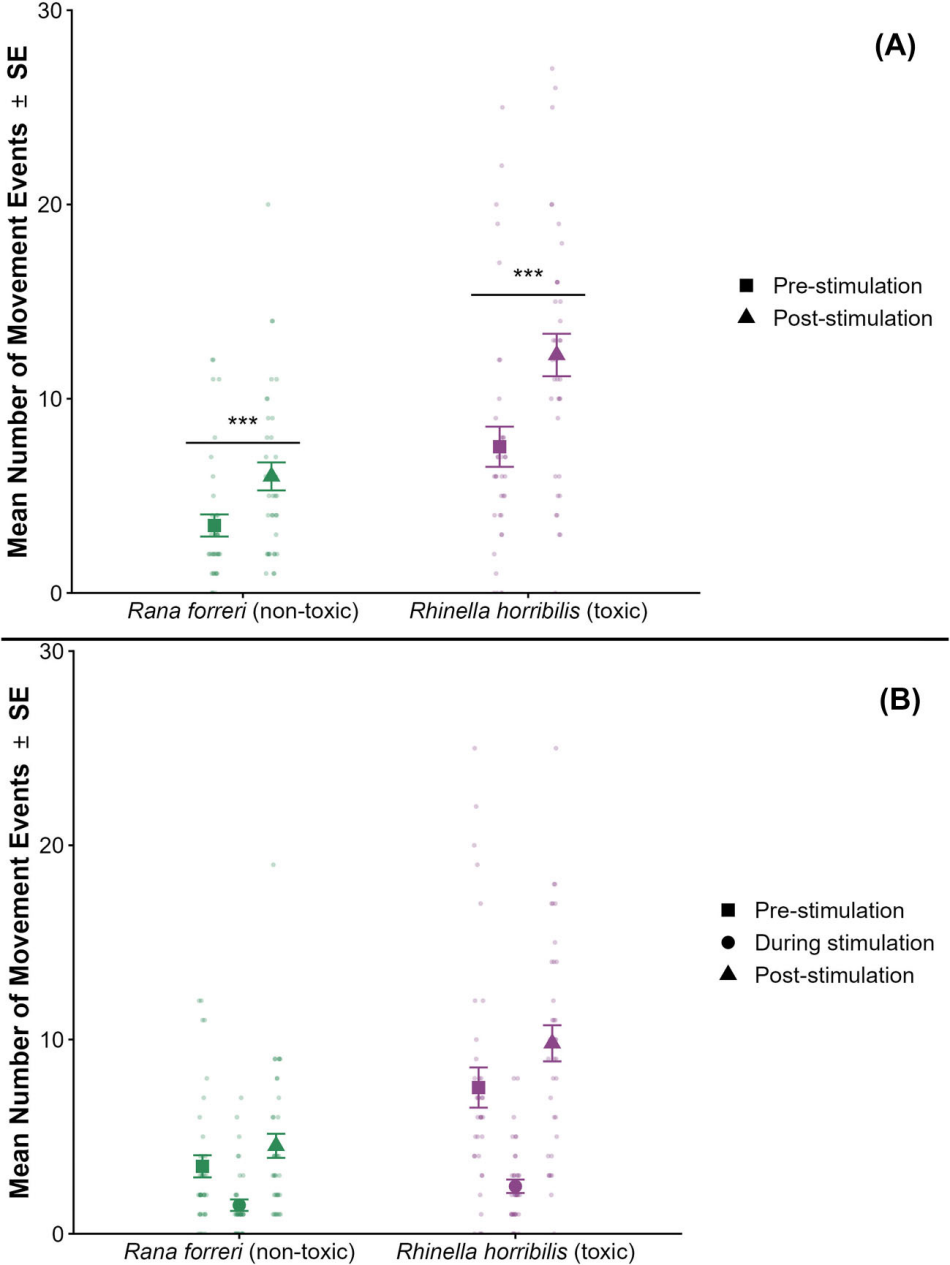
Mean number of movement events (± standard error) for *Rana forreri* (Ranidae) and *Rhinella horribilis* (Bufonidae) tadpoles across experimental time periods in Monteverde, Costa Rica. (A) Comparison of pre-stimulation (squares) and aggregated post-stimulation activity, including both during- and post-stimulation periods (triangles). Asterisks denote statistically significant differences within species, as determined by paired *t-*tests (*P < 0.05, **P < 0.01, ***P < 0.001). (B) Activity levels across pre-stimulation (squares), during-stimulation (circles), and post-stimulation (triangles) periods.

Before exposure to predation cues, *Rhinella horribilis* tadpoles ($\bar{x}$*=* 7.528, *SD* = 6.213) exhibited higher baseline activity levels than *Rana forreri* tadpoles ($\bar{x}\ $*=* 3.472, *SD* = 3.418; *t*(70) = -0.343, *P* = 0.001). After cue exposure, activity levels in both species, *R. horribilis* ($\bar{x}\ $= 12.25, *SD* = 6.561; *t*(35) = 4.911, *P* < 0.001) and *R. forreri* ($\bar{x}\ $= 6, *SD* = 4.322; *t*(35) = 4.302, *P* < 0.001), increased (Fig. 2A). When considering all three observation periods separately, activity decreased during stimulus presentation and increased during the post-stimulation period in both species (Fig. 2B).

### Activity levels across treatment groups

**Fig. 3:**
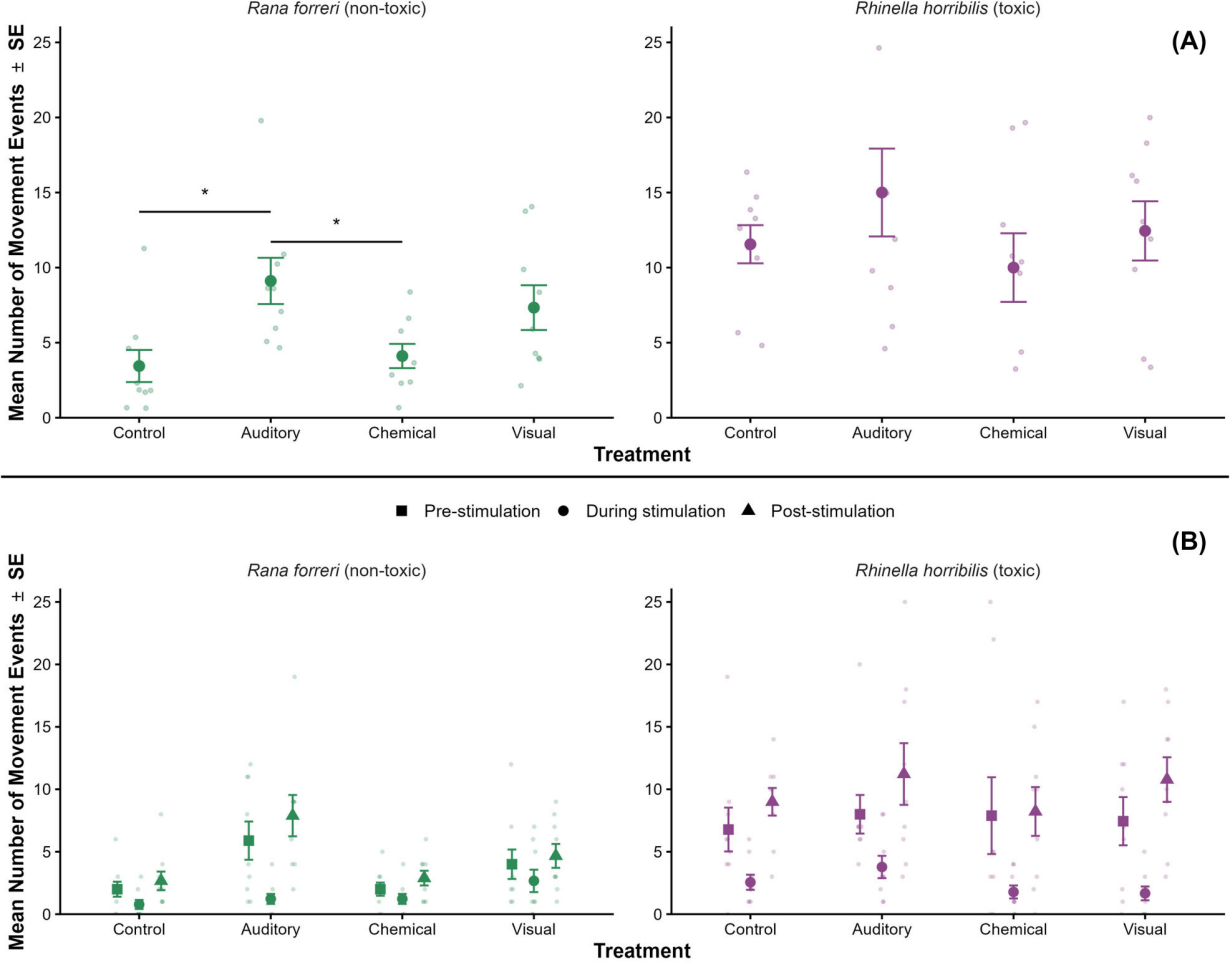
Mean number of post-stimulus movement events (± standard error) for *Rana forreri* (Ranidae; left) and *Rhinella horribilis* (Bufonidae; right) tadpoles in response to auditory, chemical, and visual predation cues, compared with control groups in Monteverde, Costa Rica. (A) Comparison of post-stimulation activity (including during- and post-stimulation periods) across treatments. Asterisks denote statistically significant pairwise differences among treatments for *R. forreri*, based on Dunn’s post hoc tests (*P < 0.05, **P < 0.01, ***P < 0.001). (B) Activity levels across pre- (squares), during- (circles), and post-stimulation (triangles) periods for each treatment.

Tadpole responses to predation cue treatments differed between species (Fig. 3A). Cane toad tadpoles exhibited similar post-simulation activity levels across all treatment groups (*F*_(3, 32)_ = 0.909, *P* = 0.448). Conversely, Forrer’s leopard frog tadpoles exhibited statistically significant activity differences across treatments (*H*(3) = 12.135, *P* = 0.007). *Rana forreri* tadpoles exposed to the auditory stimulus ($\bar{x}\ $*=* 9.111, SD= 4.622) were significantly more active than those in the control group ($\bar{x}\ $*=* 3.444, SD= 3.206; *P* = 0.011). No other pairwise comparisons were statistically significant (Chemical-Control: *P* = 0.551; Visual-Control: *P* = 0.063). A similar temporal pattern was observed across treatments in both species, with activity decreasing during stimulus presentation and increasing during the post-stimulation period prior to aggregation of during- and post-stimulation time intervals (Fig. 3B).

## Discussion

Baseline movement patterns differed between species, with *Rhinella horribilis* tadpoles exhibiting substantially higher activity levels than *Rana forreri* prior to predation cue exposure. Although activity increased in both species following stimulus introduction, *R. horribilis* showed little variation across cue types, indicating weak behavioral modulation in response to predation signals. Given that *R. horribilis* possesses toxic parotid gland secretions that function as an effective chemical defense and are a key trait in the diversification of the family Bufonidae (Wu et al. 2025), these results are consistent with the hypothesis that chemically defended tadpoles rely less on immediate behavioral responses to predation cues than undefended species.

This interpretation closely aligns with optimal escape theory, which predicts that prey initiate costly escape behaviors only when the anticipated risk of predation outweighs the energetic and opportunity costs associated with fleeing (Ydenberg and Dill 1986; Samia et al. 2015). For a highly toxic species such as *R. horribilis*, the benefits of escape may be comparatively low, resulting in weaker, delayed, or less variable behavioral responses. Similar patterns have been observed in chemically defended insects, where unpalatable species often invest less in avoidance behaviors or display bolder antipredator behavior compared to palatable congeners (Dowdy and Conner 2019; Kojima 2022).

In contrast, *Rana forreri* exhibited clear treatment-specific responses, particularly to auditory cues. Paired pre- versus post-stimulation analyses suggest that these changes reflect direct responses to experimental stimuli rather than random fluctuations in baseline activity. Although reduced activity is the most frequently documented tadpole antipredation response, increased activity can similarly function as an adaptive escape strategy when predators are perceived as imminent threats (Lima et al. 2015). In our trials, *R. forreri* tadpoles frequently exhibited rapid, erratic swimming followed by descent to the bottom of the container after exposure to kingfisher calls, consistent with a rapid startle-and-escape response intended to distance individuals from a perceived threat (Lima et al. 2015).

Across both species and all treatments, including control groups, a consistent temporal pattern was observed when assessing pre-, during-, and post-stimulation periods separately. Activity decreased during stimulus presentation and increased afterward. We interpret this pattern as evidence that post-stimulation increases in activity are influenced by factors independent of predator cues. These effects likely reflect disturbance associated with stimulus presentation or experimental handling, gradual acclimation to the experimental arena, or, in the case of experimental groups, recovery from the transient freezing or startle response observed immediately upon stimulus exposure. However, because experimental and control groups were subjected to identical conditions, these temporal effects are accounted for in treatment comparisons. Consequently, differences among treatments reflect cue-specific responses relative to this shared temporal pattern rather than absolute increases or decreases from pre-stimulus activity alone.

Auditory stimuli elicited the strongest behavioral responses in *Rana forreri*, suggesting that sound may serve as a particularly reliable indicator of predation risk in lotic environments. Acoustic signals propagate through water as particle motion and pressure waves, and in aquatic vertebrates are often detected via both the inner ear and the lateral line system, which is specialized for sensing low-frequency vibrations and water pressure changes (Montgomery et al. 2013). In lotic systems, such as those inhabited by *Rana forreri* tadpoles, aquatic prey lateral line neuromasts are often highly specialized for detecting subtle pressure changes and water movement, including disturbances generated by approaching predators or transmitted acoustic signals (Jung et al. 2025). In *Rana* tadpoles, the topography of these neuromasts is thought to enhance sensitivity to fine-scale pressure variation (Lannoo 1987), potentially enabling the precise detection of biologically relevant vibrational cues. These sensory adaptations may allow *Rana forreri* to detect predator-associated acoustic signals, such as kingfisher calls, as indicators of imminent risk. More broadly, these systems enable rapid detection and localization of potential threats, often with greater temporal resolution than visual cues (Rosenthal and Ryan 2000; Hettena et al. 2014).

Conversely, neither chemical nor visual cues elicited strong behavioral responses. Limited responses to chemical cues may reflect several factors. The gradual introduction of predator-conditioned water in our trials may have reduced cue salience relative to abrupt exposure, which has been shown to induce more vigorous responses in larval anurans (Crane and Ferrari 2017). Tadpoles at late developmental stages can also exhibit reduced responsiveness to chemical cues if perceived predation risk declines with size or if prior encounters with predators do not result in negative outcomes (Crane and Ferrari 2017). Furthermore, the absence of conspecific alarm cues, typically released from injured individuals during predation events, may have limited the strength of chemically mediated responses (Crane and Ferrari 2017; Surber-Cunningham et al. 2024). Finally, although belostomatids were present in the sampled sites, they may not represent consistently recognized predators for our species, potentially reducing the ecological relevance of the chemical cues used in this study (Polo-Cavia et al. 2010).

The lack of response to visual stimuli may reflect limitations in cue design or sensory capacity. Insufficient contrast or shadow sharpness could reduce detectability (McClure et al. 2009), and visual systems in tadpoles may not yet support fine-scale discrimination of aerial predator silhouettes. However, previous work demonstrates that even younger tadpoles can respond to visual stimuli, such as projected images or substrate color (Liu et al. 2016; Butler et al. 2024). Future experiments manipulating light intensity and shadow sharpness may help clarify the sensitivity of these species to visual predation cues.

Several limitations of this study should be considered. First, we quantified antipredation behavior using MEs as a proxy for activity, which does not capture other important dimensions of behavior, such as spatial distribution, refuge use, or movement type (Jungblut et al. 2025). Incorporating video tracking or automated behavioral analyses could provide a more comprehensive characterization of antipredation responses. Second, sampling differed between species: *Rana forreri* tadpoles were collected from multiple pools, whereas *R. horribilis* were obtained from a single site. This may have introduced confounding effects of genetic relatedness on *R. horribilis* tadpole behavior. While randomizing individuals across treatments should have distributed pond-specific effects among groups, future studies should aim for multi-pool sampling for all species studied to assess the generalizability of our findings. Third, species were maintained in water from their respective collection sites to provide conditions closely matching their natural environments, but this may have influenced cue perception, as physiochemical properties of aquatic systems affect the transmission of chemical and mechanical signals (Borghezan et al. 2021). Importantly, because treatment effects were analyzed within species, variation in water source should not affect our primary conclusions regarding species-specific responses to predation cues. Additionally, because this study was conducted within a single geographic region, our results should be interpreted as a location-specific case study. Phenotypic plasticity and local adaptation can generate population-level differences in behavior and morphology (Kraft et al. 2006), meaning that antipredation responses observed in one population may not generalize across a species’ range. Expanding this work across multiple sites will be essential to assessing the generality of these patterns. Finally, environmental variables such as temperature, which were not experimentally manipulated in this study, may influence antipredation behavior. Thermal conditions are known to affect both metabolic rates and sensory performance in ectotherms (Ladich and Maiditsch 2019), potentially altering responsiveness to predation cues. Evaluating how temperature interacts with sensory cue processing would help clarify how environmental conditions shape antipredation responses in tadpoles.

The fact that other chemically defended tadpoles have been found to rely primarily on chemical information to assess predation risk (Gazzola et al. 2024) suggests that defended species may vary widely in the sensory modalities they prioritize and that toxicity alone does not determine how prey integrate sensory information. Interspecific differences in morphology may also shape antipredation behavior. For example, *R. forreri* tadpoles are larger and may be more conspicuous to aerial predators, potentially favoring rapid and high-intensity escape responses (Johnson et al. 2015). Consistent with this, their tail morphology appears better suited for burst acceleration from rest. *Rhinella horribilis* tadpoles tend to have proportionally shorter, more muscular tails, which may facilitate sustained swimming near the substrate and reduce the need for rapid escape responses. Variation in body size, tail morphology, and swimming performance can therefore influence both predator detection and escape, potentially contributing to differences in responsiveness between species (Johnson et al. 2015). Expanding comparative analyses across species, alongside incorporating morphological-based measurements into future studies would provide a more mechanistic understanding of species-specific antipredation strategies.

Taken together, our findings highlight the selective prioritization of sensory information in shaping antipredation behavior. While *Rana forreri* exhibited cue-specific responses, particularly to auditory signals, *R. horribilis* showed relatively uniform activity across treatments, consistent with reduced reliance on behavioral defenses. Similar patterns have been documented across taxa, where different sensory modalities vary in their effectiveness for detecting predation risk (Hettena et al. 2014; Hughes et al. 2014; Lukas et al. 2021). Rather than reflecting a universal hierarchy of sensory cues, these results suggest that the prioritization of sensory information is shaped by species-specific ecological context and defensive strategy. In this way, even in systems where cues are encountered in isolation, differences in responsiveness provide insight into how prey may integrate and weigh sensory information under natural conditions. Extending this framework across life stages, environments, and taxa will be key to understanding how sensory decision-making influences predator-prey dynamics.

## Data Availability

The data underlying this article are publicly available on the Open Science Framework (OSF) repository at https://doi.org/10.17605/OSF.IO/9M6AS.

## Notes

From the symposium “Behavioral and environmental monitoring with open-source and low-cost tools” presented at the annual meeting of the Society for Integrative and Comparative Biology, January 3-7th, 2026.
